
JOURNAL INFORMATION
==============================
NLM Title Abbreviation: J Diabetes Investig
Journal ID: J Diabetes Investig
NLM Journal ID: 101520702
Journal ID: JDI

Journal of Diabetes Investigation

ISSN: 2040-1116
EISSN: 2040-1124
Publisher: Wiley

ARTICLE INFORMATION
==============================
PMCID: PMC11474521
DOI: 10.1111/jdi.13857
Article ID: JDI13857
Article version: 1
Subjects: Issue Information, Issue Information

Issue Information

Electronic publication date: 2023 Oct 18
Publication date: 2023 Nov
Volume: 14
Issue: 11
First page: 1227
Last page: 1228
Copyright: © 2023 The Authors. Journal of Diabetes Investigation published by Asian Association for the Study of Diabetes (AASD) and John Wiley & Sons Australia, Ltd
License: This is an open access article under the terms of the http://creativecommons.org/licenses/by/4.0/ License, which permits use, distribution and reproduction in any medium, provided the original work is properly cited.
License URL: https://creativecommons.org/licenses/by/4.0/

Figure count: 0
Table count: 0
Page count: 5

source-schema-version-number: 2.0
cover-date: November 2023
details-of-publishers-convertor: Converter:WILEY_ML3GV2_TO_JATSPMC version:6.4.9 mode:remove_FC converted:15.10.2024

==============================

### JDI

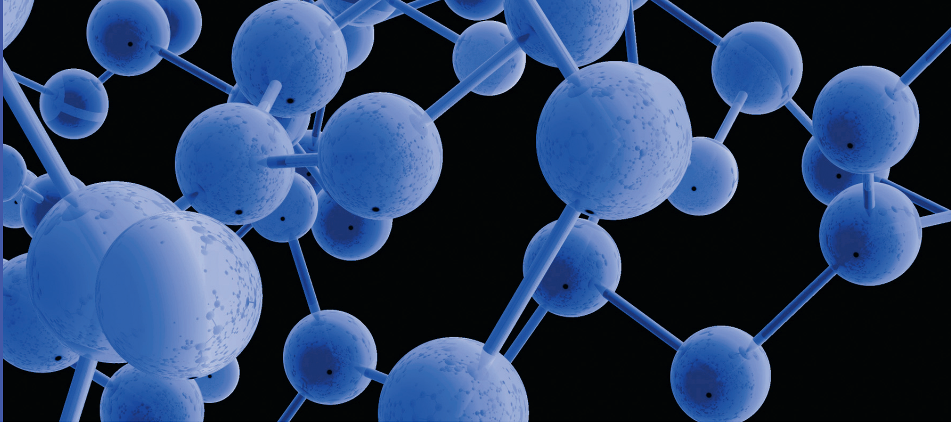

Volume 14 | Issue 11
November 2023

Journal of Diabetes Investigation
Official Journal of the Asian Association for the Study of Diabetes

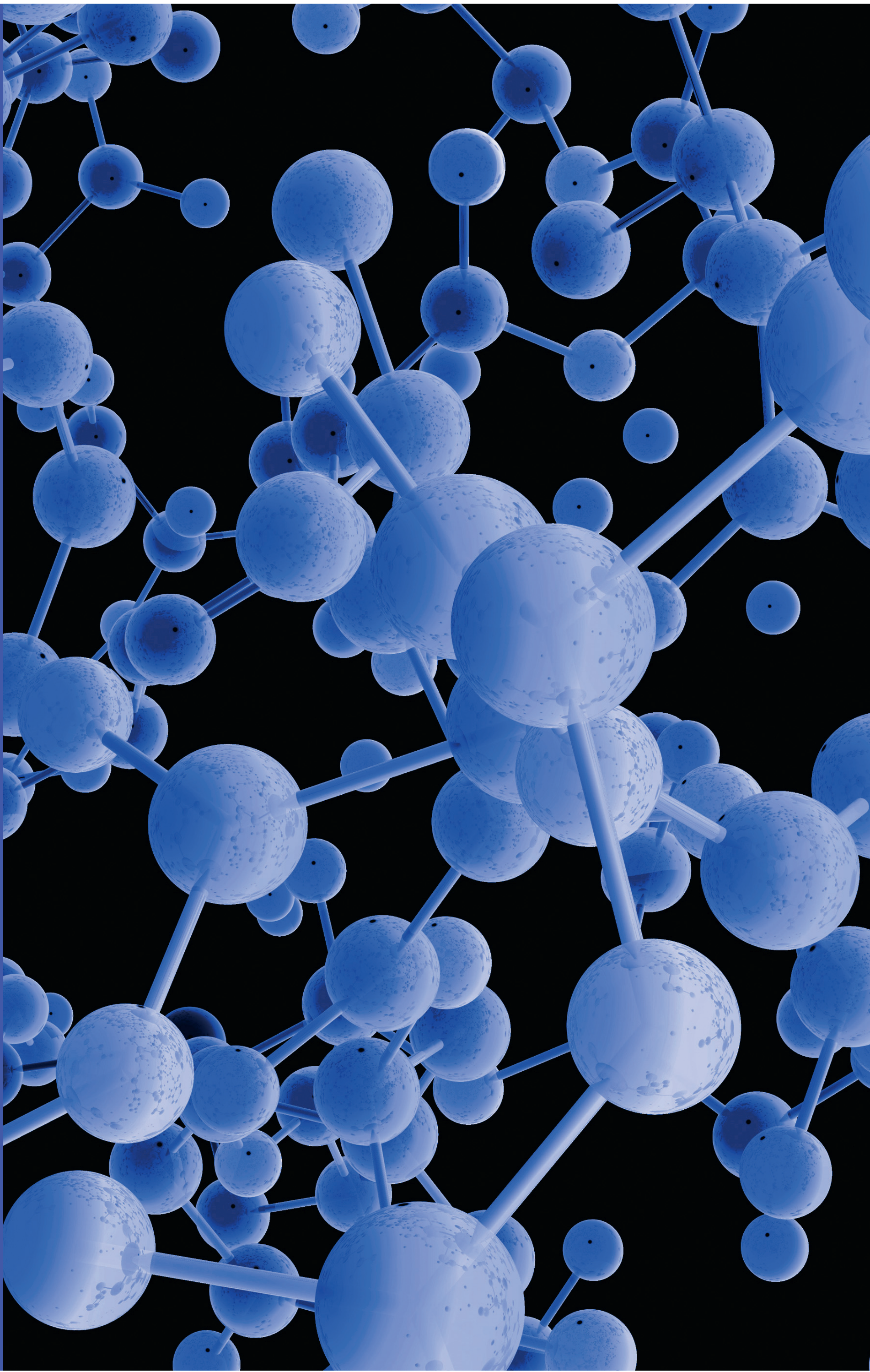

Bleeding complications and COVID-19

THE ASIAN ASSOCIATION FOR THE STUDY OF DIABETES

WILEY

### JDI Journal of Diabetes Investigation

Open access

**Official Journal of the Asian Association for the Study of Diabetes**

**Editor-in-Chief**
Nigishi Hotta, *Japan Organization of Occupational Health and Safety, Chubu Rosai Hospital, Nagoya, Japan*

**Co-Editor**
Domenico Accili, *Columbia University, New York, USA*

**Expert Editor**
Bo Ahren, *Lund, Sweden*
Young Min Cho, *Seoul, Korea*
Sean Dinneen, *Galway, Ireland*
Marc Gilbert, *Montpelier, France*
Limei Liu, *Shanghai, China*
Ronald Ma, *Hong Kong, China*
Louis Philipson, *Chicago, USA*
Wayne H-H Sheu, *Taichung, Taiwan*
Bernard Thorens, *Lausanne, Switzerland*
Daisuke Yabe, *Gifu, Japan*

**Editorial Board**
Eiichi Araki, *Kumamoto, Japan*
Xiaoping Chen, *Beijing, China*
Lee-Ming Chuang, *Taipei, Taiwan*
Masakazu Haneda, *Asahikawa, Japan*
Larry Low-Tone Ho, *Taipei, Taiwan*
Tianpei Hong, *Beijing, China*
Akihisa Imagawa, *Osaka, Japan*
Nobuya Inagaki, *Kyoto, Japan*
Linong Ji, *Beijing, China*
Weiping Jia, *Shanghai, China*
Yi-Der Jiang, *Taipei, Taiwan*
Takashi Kadowaki, *Tokyo, Japan*
Kohei Kaku, *Kurashiki, Japan*
Keizo Kanasaki, *Izumo, Japan*
Doo-Man Kim, *Seoul, Korea*
Karen Lam, *Hong Kong, China*
Hong Kyu Lee, *Gyeonggi-do, Korea*
In-Kyu Lee, *Daegu, Korea*
Moon-Kyu Lee, *Seoul, Korea*
Jiro Nakamura, *Nagakute, Japan*
Kishio Nanjo, *Wakayama, Japan*
Wataru Ogawa, *Kobe, Japan*
Yutaka Seino, *Osaka, Japan*
Shyi-Jang Shin, *Kaohsiung, Taiwan*
Hirohito Sone, *Niigata, Japan*
Kathryn Tan, *Hong Kong, China*
Yukio Tanizawa, *Ube, Japan*
Kohjiro Ueki, *Tokyo, Japan*
Jun Wada, *Okayama, Japan*
Hirotaka Watada, *Tokyo, Japan*
Jianping Weng, *Guangzhou, China*
Soroku Yagihashi, *Hirosaki, Japan*
Hiroshi Yamamoto, *Kanazawa, Japan*
Toshimasa Yamauchi, *Tokyo, Japan*
Wenying Yang, *Beijing, China*
Kazuki Yasuda, *Tokyo, Japan*
Koutaro Yokote, *Chiba, Japan*
Kun-Ho Yoon, *Seoul, Korea*
Dalong Zhu, *Nanjing, China*

**International Editorial Board**
Michael A. Brownlee, *New York, USA*
Subrata Chakrabarti, *Ontario, Canada*
Mark E. Cooper, *Prahran, Australia*
Philippe Froguel, *London, UK*
Wilfred Fujimoto, *Hawaii, USA*
Michael S. German, *San Francisco, USA*
Andrew Hattersley, *Exeter, UK*
Jean-Claude Henquin, *Brussels, Belgium*
Jens Juul Holst, *Copenhagen, Denmark*
Frank B. Hu, *Boston, USA*
C. Ronald Kahn, *Boston, USA*
George L. King, *Boston, USA*
Rayaz A. Malik, *Manchester, UK*
Michael Mauer, *Minneapolis, USA*
Carl Erik Mogensen, *Aarhus, Denmark*
Steven E. Shoelson, *Boston, USA*
Jay S. Skyler, *Miami, USA*
Paul Zimmet, *Melbourne, Australia*

**Assistant Editorial Board**
Tien-Jyun Chang, *Taipei, Taiwan*
Yoshiyuki Hamamoto, *Osaka, Japan*
Norio Harada, *Kyoto, Japan*
Yi-Jen Hung, *Taipei, Taiwan*
Chi-Min Hwu, *Taipei, Taiwan*
Hideki Kamiya, *Aichi, Japan*
Dae Jung Kim, *Suwon, Korea*
Seung-Hwan Lee, *Seoul, Korea*
Hung-Yuan Li, *Taipei, Taiwan*
Takashi Matsuzaka, *Tsukuba, Japan*
Keiko Naruse, *Aichi, Japan*
Takeshi Nishikawa, *Kumamoto, Japan*
Eung-Jung Rhee, *Seoul, Korea*
Yusuke Seino, *Aichi, Japan*
Hironori Waki, *Tokyo, Japan*

**Aims and Scope:** *Journal of Diabetes Investigation* is your core diabetes journal from Asia; the official journal of the Asian Association for the Study of Diabetes (AASD). The journal publishes original research, country reports, commentaries, reviews, mini-reviews, case reports, letters, as well as editorials and news. The journal embraces clinical and experimental research in diabetes and related areas. This includes aspects of prevention, treatment, as well as molecular aspects and pathophysiology. Translational research focused on the exchange of ideas between clinicians and researchers is also welcome.

**Address for Editorial Correspondence:**
The Editor, *Journal of Diabetes Investigation*, Nomura Fudosan Nishi Shinjuku Building, 8F 8-4-2 Nishi Shinjuku, Shinjuku-ku, Tokyo 160-0023, Japan. Tel: +81 3 4520 9009; Fax: +81 3 4520-9059 Email: JDI@wiley.com

**Copyright © 2023 Asian Association for the Study of Diabetes and John Wiley & Sons Australia, Ltd**

**Cover:** Insulin molecule © Pasieka/Science Photo Library

This journal is available online at Wiley Online Library. Visit wileyonlinelibrary.com to search the articles and register for table of contents and e-mail alerts.

**For submission instructions, and all other information visit wileyonlinelibrary.com/journal/jdi**

ISSN 2040-1124 (Online)

JDI.JEB.Nov23

WILEY

**Publisher**
*Journal of Diabetes Investigation* is published by John Wiley & Sons Australia, Ltd
Level 19, 15 William Street,
Melbourne, Victoria 3000,
Australia.
Tel: +61 (0)3 9274 3100

**Production Editor**
Jayalakshmi Mohan (email: JDIproduction@wiley.com)

**Abstracting and Indexing Services**
The Journal is indexed by Academic OneFile (GALE Cengage), Biological Abstracts (Clarivate Analytics), Biological Science Database (ProQuest), BIOSIS Previews (Clarivate Analytics), Embase (Elsevier), Health & Medical Collection (ProQuest), Health Research Premium Collection (ProQuest), Hospital Premium Collection (ProQuest), MEDLINE/PubMed (NLM), Natural Science Collection (ProQuest), ProQuest Central (ProQuest), Science Citation Index Expanded (Clarivate Analytics), SciTech Premium Collection (ProQuest), SCOPUS (Elsevier), Web of Science (Clarivate Analytics).

**Open Access and Copyright**
All articles published by *Journal of Diabetes Investigation* are fully open access: immediately freely available to read, download and share. All *Journal of Diabetes Investigation* articles accepted from 24 September 2013 are published under the terms of the Creative Commons Attribution 4.0 Unported (CC BY 4.0) license, which permits use, distribution and reproduction in any medium, provided the original work is properly cited. Articles accepted before this date were published under the agreement as stated in the final article. Copyright on any *Journal of Diabetes Investigation* article accepted after 24 September 2013, the date on which *Journal of Diabetes Investigation* became an open access journal, is retained by the author(s). Authors grant to *Journal of Diabetes Investigation* and Wiley a license to publish the article and for Wiley to identify itself as the original publisher. Authors also grant any third party the right to use the article freely as long as its integrity is maintained and its original authors, citation details and publisher are identified. Please visit https://authorservices.wiley.com/author-resources/Journal-Authors/open-access/hybrid-open-access.html for further information about Open Access.

**Use by commercial ''for-profit'' organisations (not applicable to CC BY 4.0 licensed articles)**
Use of Wiley Open Access articles for commercial, promotional, or marketing purposes requires further explicit permission from Wiley and will be subject to a fee. Requests to reproduce material from this journal content are being handled through the Rightslink® online service.

- Locate the article you wish to reproduce on Wiley Online Library (http://onlinelibrary.wiley.com)
- Navigate to the abstract page
- Click on the 'Request Permissions' link, under the 'ARTICLE TOOLS' menu
- Follow the online instructions and select your requirements from the drop down options and click on 'quick price' to get a quote
- Create a RightsLink® account to complete your transaction (and pay, where applicable)
- Read and accept our Terms & Conditions and Download your license
- For any technical queries please contact customercare@copyright.com
- For further information and to view a Rightslink® demo please visit www.wiley.com and select Rights & Permissions.
- Print reprints of Wiley Open Access articles can be purchased from corporatesales@wiley.com.

**Disclaimer**
The Publisher, the Asian Association for the Study of Diabetes, and Editors cannot be held responsible for errors or any consequences arising from the use of information contained in this journal; the views and opinions expressed do not necessarily reflect those of the Publisher, the Asian Association for the Study of Diabetes, or Editors, neither does the publication of advertisements constitute any endorsement by the Publisher, the Asian Association for the Study of Diabetes, or Editors of the products advertised.

Wiley Open Access articles posted to repositories or websites are without warranty from Wiley of any kind, either express or implied, including, but not limited to, warranties of merchantability, fitness for a particular purpose, or noninfringement.
To the fullest extent permitted by law Wiley disclaims all liability for any loss or damage arising out of, or in connection, with the use of or inability to use the content.

**Journal Customer Services:** For ordering information, claims and any enquiry concerning your journal subscription please go to https://wolsupport.wiley.com/s/contactsupport?tabset-a7d10=2 or contact your nearest office.
**Americas:** Email: cs-journals@wiley.com; Tel: +1 877 762 2974
**Europe, Middle East and Africa:** Email: cs-journals@wiley.com; Tel: +44 (0) 1865 778315; 0800 1800 536 (Germany)
**Germany, Austria, Switzerland, Luxembourg, Liechtenstein:** cs-germany@wiley.com; Tel: 0800 1800 536 (Germany)
**Asia Pacific:** Email: cs-journals@wiley.com; Tel: +65 3165 0890.
**Japan:** For Japanese speaking support, Email: cs-japan@wiley.com; .
**Visit our Online Customer Help** at https://wolsupport.wiley.com/s/contactsupport?tabset-a7d10=2

Wiley's Corporate Citizenship initiative seeks to address the environmental, social, economic, and ethical challenges faced in our business and which are important to our diverse stakeholder groups. Since launching the initiative, we have focused on sharing our content with those in need, enhancing community philanthropy, reducing our carbon impact, creating global guidelines and best practices for paper use, establishing a vendor code of ethics, and engaging our colleagues and other stakeholders in our efforts. Follow our progress at www.wiley.com/go/citizenship

VOLUME 14 – ISSUE 11 – NOVEMBER 2023

### Journal of Diabetes Investigation

Open access

### Commentary

**Delineating the transcriptional atlas for impaired insulin secretion: A window into type 2 diabetes pathophysiology**
Jian Li, Jin Yang, and Tianpei Hong **1231**

### Update

**Effects of nutrient metabolism on pancreatic β-cell mass and function: Recent findings**
Takuma Yasuda and Norio Harada **1234**

### Original Articles

### Epidemiology

**Elevated serum uric acid is a risk factor for progression to prediabetes in Japanese women: A 5-year retrospective chort study**
Masanori Shimodaira, Yu Minemura, and Tomohiro Nakayama **1237**

### Clinical Science and Care

**Efficacy, safety and tolerability of imeglimin in patients with type 2 diabetes mellitus: A meta-analysis of randomized controlled trials**
Katsuhiko Hagi, Masahiro Nitta, Hirotaka Watada, Kohei Kaku, and Kohjiro Ueki **1246**

### Short Report

**Real-world risk of cardiovascular diseases in patients with type 2 diabetes associated with sodium–glucose cotransporter 2 inhibitors in comparison with metformin: A propensity score-matched model analysis in Japan**
Takeshi Horii, Yoichi Oikawa, Akira Shimada, and Kiyoshi Mihara **1262**

### Original Articles

**Polar vasculosis is associated with better kidney outcome in type 2 diabetes with biopsy-proven diabetic kidney disease: A multicenter cohort study**
Miho Shimizu, Kengo Furuichi, Tadashi Toyama, Masayuki Yamanouchi, Junichi Hoshino, Shinji Kitajima, Akinori Hara, Yasunori Iwata, Norihiko Sakai, Yukio Yuzawa, Hiroshi Kitamura, Hiroshi Sato, Yugo Shibagaki, Yoshiki Suzuki, Noriko Uesugi, Yoshihiko Ueda, Kentaro Kohagura, Kenichi Samejima, Kazuhiko Tsuruya, Shinichi Nishi, Tomoya Nishino, Hirofumi Makino, Seiichi Matsuo, Yoshifumi Ubara, Hitoshi Yokoyama, and Takashi Wada, Research Group of Diabetic Nephropathy, the Ministry of Health, Labour and Welfare, and the Japan Agency for Medical Research and Development **1268**

**Associations of polyneuropathy with risk of all-cause and cardiovascular mortality, cardiovascular disease events stratified by diabetes status**
Kyuho Kim, Su-Nam Lee, Yu-Bae Ahn, Seung-Hyun Ko, and Jae-Seung Yun **1279**

**Machine learning for the prediction of atherosclerotic cardiovascular disease during 3-year follow up in Chinese type 2 diabetes mellitus patients**
Jinru Ding, Yingying Luo, Huwei Shi, Ruiyao Chen, Shuqing Luo, Xu Yang, Zhongzhou Xiao, Bilin Liang, Qiujuan Yan, Jie Xu, and Linong Ji **1289**

**Association between hemoglobin glycation index and non-alcoholic fatty liver disease in the patients with type 2 diabetes mellitus**
Meng Wang, Shiwei Li, Xinxin Zhang, Xin Li, and Jingqiu Cui **1303**

**Factors associated with bleeding complications in patients with coronavirus disease 2019 admitted to intensive care units: A multicenter retrospective cohort study**
Dan Imai, Emi Ushigome, Masahide Hamaguchi, Satoru Hashimoto, and Michiaki Fukui **1312**

### Case Reports

**Successful introduction of sensor-augmented pump therapy in a patient with diabetes and needle phobia: A case report**
Keiji Sugai, Junpei Shikuma, Satoshi Hiroike, Hironori Abe, and Ryo Suzuki **1318**

**Diabetic ketoacidosis due to a sensor defect of FreeStyle Libre: A case report**
Toshiki Kogai, Junko Sato, Marin Hirakata, Tatsuya Iwamoto, Kenichi Nakajima, Hiromasa Goto, Yuya Nishida, and Hirotaka Watada **1321**
